# Long-Term Conductivity Stability of Electrolytic Membranes of Scandia Stabilized Zirconia Co-Doped with Ytterbia

**DOI:** 10.3390/membranes13060586

**Published:** 2023-06-06

**Authors:** Dmitrii Agarkov, Mikhail Borik, Boris Komarov, Galina Korableva, Alexey Kulebyakin, Irina Kuritsyna, Elena Lomonova, Filipp Milovich, Valentina Myzina, Nataliya Tabachkova

**Affiliations:** 1 Osipyan Institute of Solid State Physics RAS, Academician Osipyan Str., 2, 142432 Chernogolovka, Russia; eliseevagm@issp.ac.ru (G.K.); koneva@issp.ac.ru (I.K.); 2 Moscow Institute of Physics and Technology, Institusky Lane, 9, 141700 Dolgoprudny, Russia; 3 Prokhorov General Physics Institute of Russian Academy of Sciences, Vavilova Street, 38, 119991 Moscow, Russia; borik@lst.gpi.ru (M.B.); boris.komarov.1998.2017@mail.ru (B.K.); kulebyakin@lst.gpi.ru (A.K.); lomonova@lst.gpi.ru (E.L.); vamyzina@lst.gpi.ru (V.M.); ntabachkova@misis.ru (N.T.); 4 Department of Materials Science, Moscow Polytechnic University, Bolshaya Semyonovskaya Str., 38, 107023 Moscow, Russia; philippmilovich@gmail.com; 5 Department of Materials Science of Semiconductors and Dielectrics, National University of Science and Technology «MISIS», Leninskiy Prospect, 4, 119049 Moscow, Russia

**Keywords:** zirconia membranes, SOFC, single crystal, structure, conductivity

## Abstract

The effect of high-temperature aging for 4800 h at a temperature of 1123 K on the crystal structure and the conductivity of (ZrO_2_)_0.90_(Sc_2_O_3_)_0.09_(Yb_2_O_3_)_0.01_ and (ZrO_2_)_0.90_(Sc_2_O_3_)_0.08_(Yb_2_O_3_)_0.02_ single-crystal membranes were studied. Such membrane lifetime testing is critical to the operation of solid oxide fuel cells (SOFCs). The crystals were obtained by the method of directional crystallization of the melt in a cold crucible. The phase composition and structure of the membranes before and after aging were studied using X-ray diffraction and Raman spectroscopy. The conductivities of the samples were measured using the impedance spectroscopy technique. The (ZrO_2_)_0.90_(Sc_2_O_3_)_0.09_(Yb_2_O_3_)_0.01_ composition showed long-term conductivity stability (conductivity degradation not more than 4%). Long-term high-temperature aging of the (ZrO_2_)_0.90_(Sc_2_O_3_)_0.08_(Yb_2_O_3_)_0.02_ composition initiates the t″ → t′ phase transformation. In this case, a sharp decrease in conductivity of up to 55% was observed. The data obtained demonstrate a clear correlation between the specific conductivity and the change in the phase composition. The (ZrO_2_)_0.90_(Sc_2_O_3_)_0.09_(Yb_2_O_3_)_0.01_ composition can be considered a promising material for practical use as a solid electrolyte in SOFCs.

## 1. Introduction

One of the current problems of economic development is the achievement of clean and efficient production of electricity from renewable sources with minimal emissions of pollutants and carbon dioxide into the atmosphere. Solid oxide fuel cells (SOFCs) play an important role in achieving this goal and enable the transition from fossil fuels to other renewable energy sources. Materials based on zirconium dioxide are well-known solid electrolytes [1,2,3] and are widely used as electrolytic membranes in solid oxide fuel cells [4,5]. Long-term exposure of electrolytic membranes to elevated operating temperatures can lead to changes in their functional characteristics. Aging processes in solid electrolytes, which manifest themselves in a change in their electrical conductivity during isothermal annealing, have been studied in solid solutions based on zirconium dioxide stabilized by oxides of various rare earth elements [6,7,8,9]. It has been shown that, in the general case, there are three independent reasons for the aging of solid electrolytes based on ZrO_2_: decomposition of the solid solution with the formation of new phases; ordering of cations and oxygen in the crystal lattice of a solid solution; and change in electrical conductivity of grain boundaries. The processes of aging of solid electrolytes of zirconium dioxide stabilized by yttrium or scandium oxides have been studied in great detail. To a lesser extent, the long-term effect of temperature on solid solutions of zirconium dioxide stabilized by yttrium or scandium oxides and additionally doped with other oxides of rare earth or transition elements have been studied.

Yttria-stabilized zirconia (YSZ) is still widely used as a solid electrolyte. The aging of YSZ solid electrolytes was considered in [10,11,12,13,14,15,16,17]. When studying YSZ ceramic samples, it was noted that in addition to volumetric degradation, there is also a change in the resistance of grain boundaries, which, in combination with a change in bulk conductivity, affects the degradation of the total conductivity of solid solutions. The degradation in conductivity was observed to the greatest extent at a Y_2_O_3_ content of less than 9 mol%. It has been shown that the degree of degradation of the solid solutions decreases with an increase in the Y_2_O_3_ content, and no degradation has been observed at a Y_2_O_3_ concentration of ≥10 mol%. In a number of cases, annealing of ceramic membranes at 1000 °C for 1000–5000 h led to an increase in the volume resistance at a constant intergrain resistance. For tetragonal solid solutions, a decrease in the electrical conductivity of ceramic samples during such annealing occurred due to an increase in both intergrain and bulk resistance [10]. The authors of this work came to the conclusion that the successive decrease in conductivity during thermal cycling up to 1000 °C is associated with the formation of a monoclinic phase at the grain boundaries. In many works, a significant decrease in the electrical conductivity of membranes has been noted at the initial stage of annealing, after which there are practically no changes in the electrical conductivity. Thus, the electrical conductivity of 8YSZ decreased from 0.16 to 0.11 S/cm during the first 1300 h of annealing, and then, up to 2700 h of annealing, no further decrease in conductivity was observed [16]. A similar characteristic of the change in the conductivity of 8YSZ samples upon annealing at 875 °C was noted in [17]. Such a change in the electrical conductivity of 8YSZ solid solutions during long-term high-temperature exposure was associated with the release of impurities at the grain boundaries.

Of the electrolytic membranes based on zirconium dioxide, ZrO_2_–Sc_2_O_3_ (ScSZ) solid solutions have the highest conductivity; therefore, much attention has been paid to the study of the processes of high-temperature degradation of the conductivity of these materials [16,18,19]. It was shown that annealing at 1000 °C for 6000 h of 8ScSZ ceramic samples with a cubic fluorite structure led to a significant degradation in conductivity, while no change in conductivity was observed for rhombohedral samples of 11ScSZ and 12ScSZ [18]. However, membranes containing a rhombohedral phase are subject to a martensitic cubic ↔ rhombohedral phase transition at ~550 °C, leading to a change in the volume of the material, which can cause the destruction of the electrolytic cell.

In order to improve the long-term stability of the phase composition and transport characteristics of solid electrolytes based on zirconium dioxide, additional doping is often used. As a rule, yttrium oxide or oxides of other rare earth elements are introduced into zirconium dioxide stabilized by scandium oxide, which has a high oxygen ion conductivity. The study of the aging of such ceramic materials has been carried out in a number of works [13,16,19,20].

The aging of ceramic samples of the Y_2_O_3_–Sc_2_O_3_–ZrO_2_ system with total scandium and yttrium oxide concentrations of 8 mol% and 9 mol% was studied in [8,9], respectively. For these compositions, a decrease in the electrical conductivity was observed upon prolonged exposure for 1700 h at a temperature of 1000 °C. The authors explained this change in conductivity by the redistribution of Y_2_O_3_ in the sample, which led to the precipitation of the tetragonal ZrO_2_ phase in the bulk of the cubic matrix.

In [20], a study was made of the long-term conductivity stability of ceramic samples of ZrO_2_–Sc_2_O_3_ solid solutions additionally doped with 1 mol% Yb_2_O_3_ at a temperature of 600 °C under oxidizing and reducing conditions. After 2000 h of exposure to air and under reducing conditions, the decrease in the conductivity of the samples was 9.0 and 12.0%, respectively. It was shown that during the annealing process, the volume resistance of the grains remained almost stable, while the intergrain resistance contributed to an increase in the total resistivity in both atmospheres.

Thus, for ternary systems based on zirconium dioxide, the difficulty in determining the mechanisms of aging is associated both with the uncertainty of the phase boundaries in the phase diagrams and with the separation of the contributions to the overall degradation of the solid electrolyte from the degradation of bulk conductivity and conductivity at grain boundaries.

In this work, the process of aging of the anionic conductivity of membranes of solid solutions of zirconium dioxide stabilized by scandium oxide and ytterbium oxide at a temperature of 1123 K with exothermic exposure for 4800 h was studied. The stability of the phase composition and specific conductivity of the single-crystal membranes were estimated as a function of the concentration of Yb_2_O_3_.

## 2. Materials and Methods

**Figure 1:**
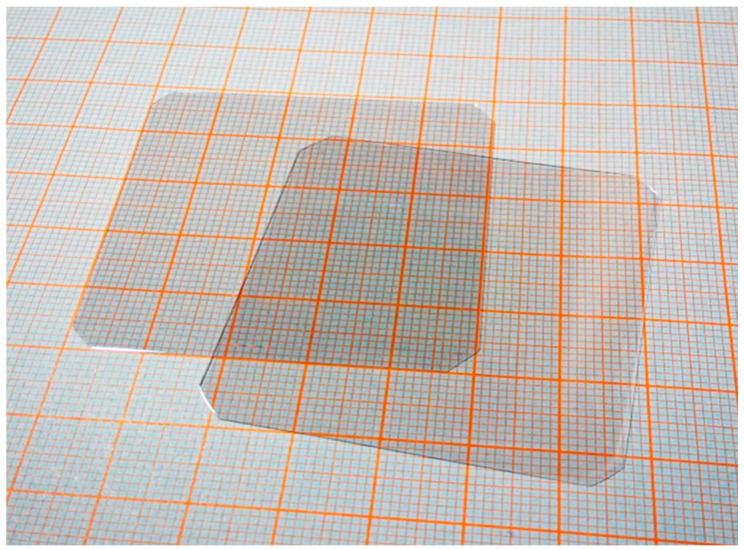
Single-crystal membranes made from cubic ZrO_2_ crystals stabilized with Y_2_O_3_, 50 × 50 mm^2^ in size.

(ZrO_2_)_0.90_(Sc_2_O_3_)_0.09_(Yb_2_O_3_)_0.01_ and (ZrO_2_)_0.90_(Sc_2_O_3_)_0.08_(Yb_2_O_3_)_0.02_ single crystals were grown by directional melt crystallization using the skull melting technique [21]. A high-frequency generator with a frequency of 5.28 MHz and an output power of 63 kW was used as a heating source. The crucible was a structure of water-cooled copper tubes mounted on a dielectric base. The crucible’s diameter was 120 mm. The starting materials used were zirconium, scandium, and ytterbium oxide powders with a purity of at least 99.99%. Powders in the required proportions were mechanically mixed and loaded into the crucible. The mass of the initial mixture was ~5 kg. Directional crystallization of the melt was carried out by lowering the crucible relative to the inductor at a rate of 10 mm/h. As a result, a polycrystalline ingot was obtained, consisting of several tens of individual single crystals. Typical crystal sizes were 40 mm in length and 20 mm in diameter. It should be noted that with an increase in the mass of the melt, this method of growth makes it possible to obtain crystals of up to 100 mm in diameter. Figure 1 shows single-crystal membranes made from cubic ZrO_2_ crystals stabilized with Y_2_O_3_, 50 × 50 mm^2^ in size.

To carry out structural and electrophysical studies, 0.5 mm thick plates were cut from the central part of single crystals perpendicular to the direction of growth. For the structural analysis, the plates were oriented so that the <100> crystallographic direction was perpendicular to the sample surface.

The phase composition and crystal lattice parameters were determined by X-ray diffraction using a Bruker D8 diffractometer with a LYNXEYE position-sensitive detector. Monochromatic CuKα radiation with a wavelength λ = 0.154 nm was used. Diffractograms were taken in a symmetrical 2θ-ω shooting scheme. The scan time per step was fixed at 1 s with a step size of 0.02° in the 2θ range of 20° to 140°. The grating parameters were measured from the diffraction maximum at large angles of 2θ~130°. The phase composition of the crystals was also studied by Raman spectroscopy in the frequency range 80–1000 cm^−1^. A laser with a wavelength of 532 nm was used as the excitation source [22,23,24,25].

The study of the electron transport characteristics of anion-conducting membranes was carried out using the four-contact method on alternating currents. The frequency dependences of the complex impedance Z*(ω) = Z′(ω) + jZ″(ω) were measured with a Solartron SI 1260 spectrometer in the frequency range 0.1–4 MHz, and the amplitude of the applied AC signal was 24 mV. The measurements were carried out in an air atmosphere in the temperature range of 350–900 °C with a step of 50 degrees while holding at each temperature for 30–40 min. The samples for measurements were membranes 7 × 7 mm^2^ in size and 0.5 mm thick, on which platinum paste was applied as electrodes on both sides. On top of the same paste, platinum wires were glued as current collectors. The platinum paste was annealed at a temperature of 950 °C for 1 h. The value of the volume resistance of the samples (R_b_) in the low-temperature range (623–723 K) was calculated within the framework of the model of the equivalent electrical circuit (R_b_-CPE_b_) (R_electrode_–CPE_electrode_). In the high-temperature range (723–1173 K), the LR_b_ (R_electrode_–CPE_electrode_) model was used, where R_electrode_ is the resistance at the electrode/electrolyte interface, CPE_electrode_ is a constant phase element that characterizes the processes at the electrode interface, and L is the inductance of the current collectors. Impedance spectra were processed using the ZView (version 2.8) program (Scribner Associates, Inc., Southern Pines, NC, USA). The specific conductivity of the membranes σ was calculated from the data obtained by processing the impedance spectra, taking into account the geometric parameters of the samples as σ = 1/R_b_(l/S), where l is the sample thickness and S is the contact area.

## 3. Results and Discussion

The study of high-temperature conductivity degradation was carried out on single-crystal membranes of two compositions with a total concentration of stabilizing oxides (Sc_2_O_3_ + Yb_2_O_3_) equal to 10 mol%: (ZrO_2_)_0.90_(Sc_2_O_3_)_0.09_(Yb_2_O_3_)_0.01_ and (ZrO_2_)_0.89_(Sc_2_O_3_)_0.09_(Yb_2_O_3_)_0.02_, hereinafter denoted as 9Sc1YbSZ and 8Sc2YbSZ, respectively. For comparison, previously published data on (ZrO_2_)_0.9_(Sc_2_O_3_)_0.1_(10ScSZ) crystals are also given [26]. Such a comparison seems to be correct because these crystals were obtained using the same technology (directional crystallization of the melt) from identical starting materials in the same temperature and time technological regimes.

All the crystals had a columnar shape after growth, which is typical for this method of growth. The 10ScSZ crystals were visually inhomogeneous in volume and opalescent. The substitution of even 1 mol% Sc_2_O_3_ for Yb_2_O_3_ (9Sc1YbSZ) led to homogeneous transparent crystals. The 8Sc2YbSZ crystals did not differ in appearance from the 9Sc1YbSZ crystals.

An X-ray diffraction study of the crystal structure showed that the 10ScSZ crystals were two-phase and contained cubic and rhombohedral phases, with the crystal lattice parameter of the cubic phase *a* being 5.091 Å [26]. The as-grown 9Sc1YbSZ and 8Sc2YbSZ crystals had cubic structures with parameters *a* equal to 5.094 and 5.099 Å, respectively. Thus, the successive replacement of scandium oxide by ytterbium oxide in the series 10ScSZ → 9Sc1YbSZ → 8Sc2YbSZ leads to a monotonic increase in the lattice parameter *a*, which indicates the complete solubility of ytterbium oxide with the formation of a solid solution. This dependence seems to be regular since the Sc^3+^ ion (ionic radius 0.87 Å) is replaced by a larger Yb^3+^ ion (ionic radius 0.985 Å).

**Figure 2:**
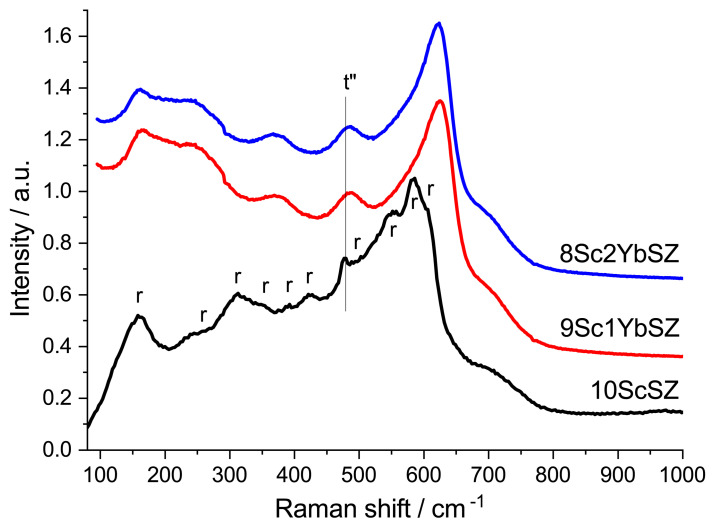
Raman spectra of 9Sc1YbSZ, 8Sc2YbSZ, and 10ScSZ [26] membranes before aging.

The crystal structures of the samples were also studied by Raman spectroscopy. Figure 2 shows the Raman spectra of the as-grown 9Sc1YbSZ and 8Sc2YbSZ crystals, as well as a 10ScSZ crystal. The spectra of the 9Sc1YbSZ and 8Sc2YbSZ crystals were similar and had a band at 480 cm^−1^, which is characteristic of the tetragonal t″ phase [27]. The Raman spectrum of 10ScSZ crystals contains bands corresponding to a mixture of rhombohedral and tetragonal t″ phases [26]. Thus, the results obtained by X-ray diffraction and Raman spectroscopy confirm previously published data on the positive effect of the addition of Yb_2_O_3_ on the stabilization of the cubic (pseudocubic t″) phase [19].

**Figure 3:**
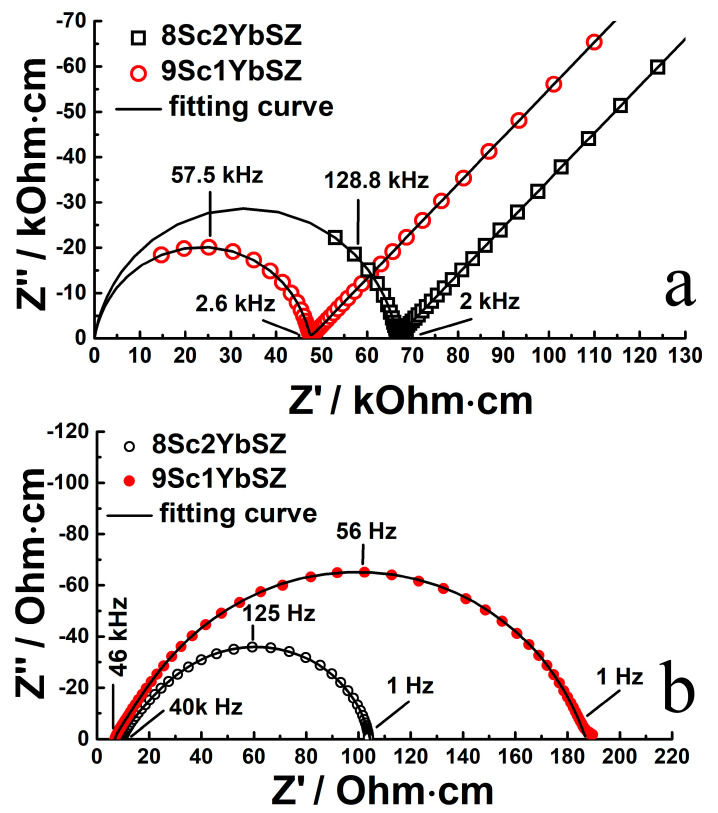
Impedance spectra in Nyquist coordinates for 9Sc1YbSZ and 8Sc2YbSZ single-crystal solid electrolyte membranes at 624 K (**a**) and 1123 K (**b**).

Figure 3 shows typical impedance spectra in Nyquist coordinates for single-crystal solid electrolyte membranes with compositions of 9Sc1YbSZ and 8Sc2YbSZ, measured at temperatures of 624 and 1123 K, respectively. In the presented impedance hodographs for the low-temperature range (624–723 K), two regions can be distinguished: a high-frequency region corresponding to the bulk impedance response of the sample under study and a low-frequency region corresponding to the electrode impedance of the electrode/electrolyte interface. The high-frequency region of the impedance spectrum (at frequencies above ~2 kHz) is an arc of a circle, with the center lying almost on the real resistance axis. The radius of this circular arc is R_b_/2, where R_b_ corresponds to the volume resistance of the solid electrolyte membrane. To describe it, an equivalent electrical circuit (R_b_-CPE_b_) was used, which is a resistor (R_b_) connected in parallel with a constant phase element (CPE_b_). The value of the exponential index CPE_b_ was about 0.85–0.95. The low-frequency region of the impedance spectrum is almost a straight line at an angle close to 45° with respect to the real resistance axis. This indicates the occurrence of the process of linear semi-infinite diffusion, which is due to the slow kinetics of oxygen exchange at the electrode/electrolyte interface in the region of low temperatures.

With an increase in temperature, a decrease in the diameter of the circle is observed, and a smaller part of the high-frequency arc remains, while the low-frequency component of the impedance, which is responsible for the electrode reactions, tends toward the reversibility of the oxygen exchange at the interface. In the high-temperature range (>873 K), the impedance spectrum is a combination of an inductive “tail”, which manifests itself in the high-frequency region of the spectrum and corresponds to the inductive contribution from the supply wires, and a circular arc, which characterizes the reversible electrode processes at the electrode/electrolyte interface. In this case, the volume resistance of the electrolyte (R_b_) can be calculated from the left-hand intersection of the impedance response arc with the real resistance axis. As an example, the figure shows the typical impedance spectra at 1123 K.

**Figure 4:**
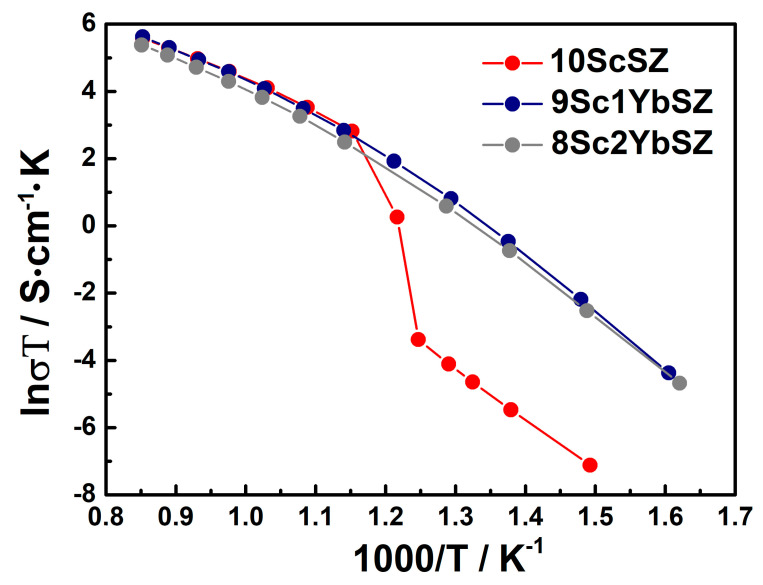
Arrhenius plots for the conductivities of the as-grown 9Sc1YbSZ, 8Sc2YbSZ, and 10ScSZ [26] crystals.

Figure 4 shows the temperature dependences of the conductivities of as-grown 9Sc1YbSZ, 8Sc2YbSZ, and 10ScSZ crystals in Arrhenius coordinates. The temperature dependence of the conductivity of a 10ScSZ crystal in the temperature range of 833–913 K exhibits a kink due to the rhombohedral–cubic polymorphic transition [26]. When scandium oxide is replaced by ytterbium oxide (samples 9Sc1YbSZ and 8Sc2YbSZ), no such break is observed because, according to the Raman spectroscopy data, these crystals have the structure of a pseudocubic t″ phase. The temperature dependencies of the conductivities of the 9Sc1YbSZ and 8Sc2YbSZ samples have a noticeable curvature. A similar change in slope, previously observed in other ZrO_2_–Sc_2_O_3_ systems, is associated with the destruction of local defect complexes at high temperatures [28]. The values of the high-temperature electrical conductivity of the 10ScSZ, 9Sc1YbSZ, and 8Sc2YbSZ crystals were 0.224, 0.235, and 0.18 S/cm, respectively, at 1173 K. Thus, the substitution of 1 mol% Sc_2_O_3_ for Yb_2_O_3_ leads to the stabilization of the cubic (pseudocubic) phase without decreasing the high-temperature conductivity of the material. However, upon further substitution of Sc_2_O_3_ with Yb_2_O_3_, a decrease in conductivity was observed in the high-temperature region.

To carry out long-term conductivity measurements, samples with platinum electrodes placed in a tubular resistance furnace were heated to a temperature of 1123 K. Further, periodic measurements of conductivity were carried out in-situ with an interval of 24 to 48 h. Then, the sample was cooled to 623 K, and repeated studies of electrical conductivity were carried out in the temperature range of 623–1173 K.

**Figure 5:**
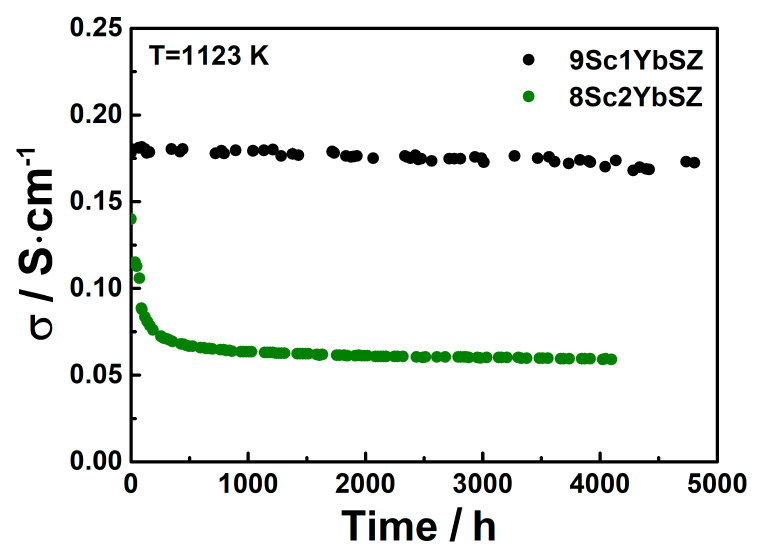
Conductivity degradation of 9Sc1YbSZ and 8Sc2YbSZ solid electrolytes at 1123 K.

Figure 5 shows the changes in the specific conductivities of the 9Sc1YbSZ and 8Sc2YbSZ electrolytic membranes during aging at 1123 K.

As can be seen from Figure 5, after 4800 h of exposure at 1123 K, the conductivity of the 9Sc1YbSZ electrolytic membrane changed from the initial value from 0.180 to 0.172 S/cm, which corresponds to a degradation degree of 4%. The nature of the change in the conductivity of the 8Sc2YbSZ sample differs sharply from the change in the conductivity of 9Sc1YbSZ. Thus, during the first 500 h of aging, there is a sharp decrease in electrical conductivity from 0.142 to 0.066 S/cm, which is 54% of the initial value. With further exposure of the sample at 1123 K, a gradual decrease in conductivity to a value of 0.06 S/cm is observed. Thus, the total drop in conductivity after holding at 1123 K for 4200 h was 58%.

**Figure 6:**
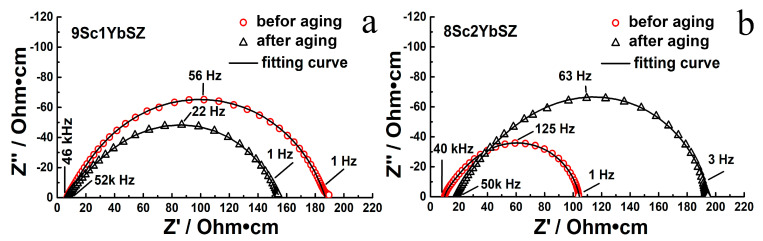
Impedance spectra for 9Sc1YbSZ (**a**) and 8Sc2YbSZ (**b**) single-crystal solid electrolyte membranes at 1123 K before and after aging.

Figure 6 shows impedance spectra for 9Sc1YbSZ and 8Sc2YbSZ single-crystal solid electrolyte membranes before and after aging, measured at 1123 K.

**Figure 7:**
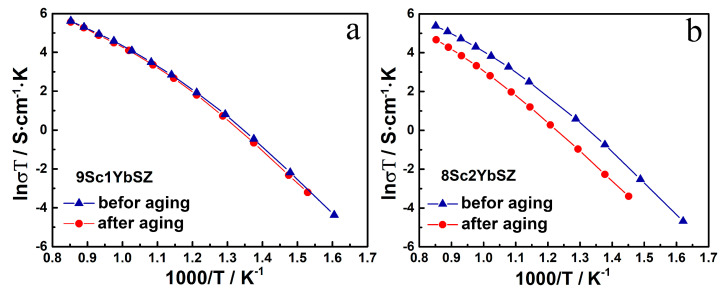
Arrhenius plots for the conductivity of 9Sc1YbSZ (**a**) and 8Sc2YbSZ (**b**) membranes before and after aging.

Figure 7 shows the temperature dependencies of the conductivities of the 9Sc1YbSZ and 8Sc2YbSZ membranes after aging.

As can be seen from the data presented in Figure 7 for 9Sc1YbSZ membranes, the high-temperature conductivity values before and after aging are comparable, and a slight decrease in conductivity after aging for this composition is observable only in the low-temperature region (643–833 K). For 8Sc2YbSZ membranes, a decrease in conductivity was clearly seen after aging over the entire temperature range.

It is interesting to compare the obtained results with the previously published data on conductivity degradation in the Yb_2_O_3_-Sc_2_O_3_-ZrO_2_ system. Thus, in 1Yb_2_O_3_-10Sc_2_O_3_-89ZrO_2_ samples after annealing at 600 °C for 500 h, the drop in conductivity was <1% [28]. At the same time, for the compositions xYb_2_O_3_-(12-x)Sc_2_O_3_-88ZrO_2_ (0 ≤ x ≤ 5), after annealing at 600 °C for 550 h, the conductivity degradation was 17–26%, depending on the composition [29]. An in-situ study of the degradation of the conductivity of ceramic samples xYb_2_O_3_-ySc_2_O_3_-(100-x-y)ZrO_2_ (x = 0; 1; y = 8; 9 mol%) at 650 °C for 2000 h showed that the most significant decrease in the conductivity occurs in the first 650 h. After that, the rate of degradation dropped sharply. The maximum conductivity degradation (10–16%) after 2000 h of aging was observed for compositions with a total concentration of stabilizing oxides equal to 8 mol%. Compositions containing 9 mol% of stabilizing oxides were relatively stable (conductivity degradation was 3%) [30]. Aging of (ZrO_2_)_0.90_(Sc_2_O_3_)_0.09_(Yb_2_O_3_)_0.01_ samples at 900 °C for 500 h led to a decrease in conductivity by 20% [31]. A direct comparison of the obtained results with the literature data is not possible because the aging of the samples was carried out in different temperature–time regimes, the conductivity was measured at different temperatures, and the initial samples were synthesized under various technological regimes and may have different crystal structures, even with the same chemical compositions.

To establish the possible causes of the degradation of conductivity, the samples after aging were re-examined by X-ray diffraction and Raman spectroscopy.

**Figure 8:**
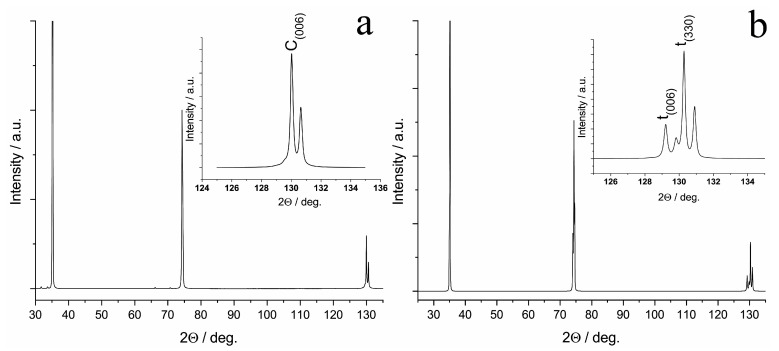
Diffraction pattern of the specimen cut from the 8Sc2YbSZ crystal perpendicular to the <001> axis before (**a**) and after (**b**) aging.

In the diffraction pattern of the 9Sc1YbSZ sample after high-temperature exposure for 4800 h, reflections were present only from the cubic modification of ZrO_2_, i.e., the 9Sc1YbSZ membranes retained their phase composition after aging. The lattice parameter of the cubic structure after aging did not change within the measurement error and was equal to 5.094 Å. In contrast to the 9Sc1YbSZ composition, a change in the phase composition was observed during the long-term high-temperature exposure of the 8Sc2YbSZ membranes; namely, the aging of the 8Sc2YbSZ samples led to a phase transformation of the initial cubic phase into a tetragonal (t′) phase (Figure 8). The lattice parameters of the t′ phase were *a* = 3.602 Å and *c* = 5.116 Å, which corresponded to a degree of tetragonality (*c*/√2*a*) equal to 1.004.

**Figure 9:**
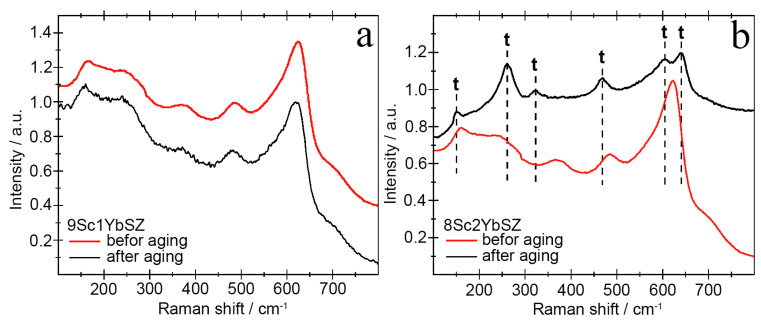
Raman spectra of 9Sc1YbSZ (**a**) and 8Sc2YbSZ (**b**) membranes before and after aging.

The results of studying the phase composition of the membranes after aging using Raman spectroscopy were in good agreement with the X-ray diffraction data. Figure 9 shows the Raman spectra of the 9Sc1YbSZ and 8Sc2YbSZ samples before and after aging.

As follows from the data presented in Figure 9a, long-term high-temperature holding of 9Sc1YbSZ samples does not lead to noticeable changes in the crystal structure. The shift of the main lines in the Raman spectra before and after aging was no more than 5 cm^−1^. Aging of the 8Sc2YbSZ membranes led to significant changes in the Raman spectra (Figure 9b). After aging, the ~161 cm^−1^ band shifted to a shorter wavelength region, 155 cm^−1^, which may be due to a decrease in the O_I_-Zr-O_I_ and Zr-O_I_-Zr bond lengths. The mode with a frequency of 242 cm^−1^ undergoes a softening during a long working life (an increase in the wave number up to 261 cm^−1^), which corresponds to the vibrations of Zr-O_II_. In the Raman spectrum after aging, the bands at 205, 362, and 585 cm^−1^, corresponding to the cubic modification of ZrO_2_, disappeared, and the bands at 261, 324, 609, and 643 cm^−1^, corresponding to the tetragonal phase of ZrO_2_, appeared. Separately, it is worth highlighting the change in the position of the ~488 cm^−1^ band, which corresponds to the pseudocubic t″-phase in the as-grown single crystal. After long-term high-temperature exposure, this vibrational mode shifts to ~469 cm^−1^, which corresponds to the tetragonal (t′) phase. Thus, during the aging of the single-crystal 8Sc2YbSZ membrane, the transformation of the pseudocubic t″ phase into the tetragonal t′ phase is observed.

**Figure 10:**
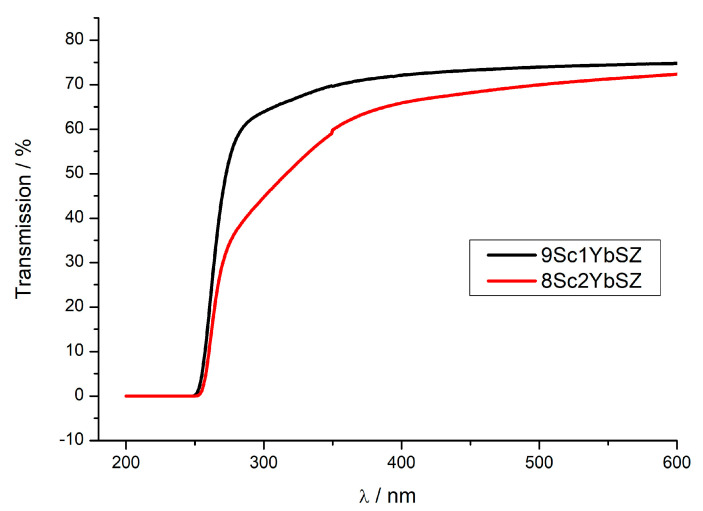
Transmission spectra of 9Sc1YbSZ and 8Sc2YbSZ crystalline membranes after aging at 1123 K for 4800 and 4200 h, respectively.

As it was noted earlier, the initial membranes were single-crystal and transparent in the visible region of the spectrum. Figure 10 shows the transmission spectra of 0.5 mm thick 9Sc1YbSZ and 8Sc2YbSZ membranes after long-term high-temperature exposure. As can be seen from the figure, after aging, a decrease in the transmission of the 8Sc2YbSZ sample relative to that of 9Sc1YbSZ is observed, which is especially obvious in the short-wavelength region of the spectrum. This type of transmission spectrum is characteristic of transparent ceramics, in which the decrease in transmission is caused by optical losses due to scattering at the pores and grain boundaries [32]. We believe that the source of the observed optical losses in the 8Sc2YbSZ sample is light scattering at the boundaries of the twins of the tetragonal t′ phase. As was found earlier, twinning of the tetragonal phase is a characteristic feature of stabilized zirconia crystals grown by directional crystallization of the melt. However, such twinning does not affect the conductivity of the crystals [33].

Thus, the analysis of the obtained data on the effect of long-term high-temperature exposure on the crystal structure and conductivity of the 9Sc1YbSZ and 8Sc2YbSZ single-crystal membranes allowed us to draw the following conclusions. After aging, the 9Sc1YbSZ samples retain their original pseudocubic structure of the t″ phase, which indicates effective stabilization of the crystal structure upon substitution of 1 mol% Sc_2_O_3_ for Yb_2_O_3_. Such phase stability, apparently, also leads to the stability of the conductivity after 4800 h of exposure at a temperature of 1123 K (the degradation of conductivity is no more than 4%). At the same time, aging of the 8Sc2YbSZ samples leads to significant changes in both the crystal structure and oxygen ionic conductivity. Long-term high-temperature holding of the 8Sc2YbSZ composition initiates the t″ → t′ phase transformation. In this case, a sharp decrease in conductivity was observed, amounting to 55%. The data obtained demonstrate a clear correlation between the specific conductivity and the change in the phase composition.

## 4. Conclusions

(ZrO_2_)_0.90_(Sc_2_O_3_)_0.09_(Yb_2_O_3_)_0.01_ and (ZrO_2_)_0.90_(Sc_2_O_3_)_0.08_(Yb_2_O_3_)_0.02_ single crystals were grown by directional melt crystallization using the skull melting technique. The grown crystals had a pseudocubic t″-phase structure. A comparison with (ZrO_2_)_0.90_(Sc_2_O_3_)_0.10_ crystals grown by the same method and consisting of a mixture of cubic and rhombohedral phases showed that the substitution of even 1 mol% Sc_2_O_3_ for Yb_2_O_3_ leads to the formation of single-phase crystals with a pseudocubic t″-phase structure without reducing the high-temperature conductivity of the material. Upon further substitution of Sc_2_O_3_ with Yb_2_O_3_, this structure was retained, and a decrease in conductivity was observed in the high-temperature region.

The effect of long-term high-temperature exposure on the crystal structure and oxygen ionic conductivity of the 9Sc1YbSZ and 8Sc2YbSZ single-crystal membranes was studied. The samples were kept for up to 4800 h at 1123 K. Aging of the 9Sc1YbSZ samples led to a weakly pronounced monotonic change in the high-temperature conductivity (the degree of degradation after 4800 h of aging did not exceed 4%). However, no structural changes were found. The nature of the change in the conductivity of the 8Sc2YbSZ sample noticeably differs from the change in the conductivity of 9Sc1YbSZ. A sharp decrease in ionic conductivity (by ~55% of the initial value) was observed during the first 500 h of aging. Further, a gradual decrease in conductivity was observed. Aging of the 8Sc2YbSZ composition led to the transformation of the initial pseudocubic t″ phase into the tetragonal t′ phase.

Thus, we can conclude that the substitution of Sc_2_O_3_ for Yb_2_O_3_ in compositions containing a total concentration of stabilizing oxides (Sc_2_O_3_ + Yb_2_O_3_) equal to 10 mol% effectively suppresses the formation of the rhombohedral phase and leads to the stabilization of the pseudocubic (cubic) structure. However, the obtained aging data showed that an increase in the Yb_2_O_3_ content to 2 mol% led to a decrease in the stability of the highly symmetrical phase. The composition (ZrO_2_)_0.90_(Sc_2_O_3_)_0.09_(Yb_2_O_3_)_0.01_ can be considered as a promising material for practical use as a solid electrolyte in SOFCs.

## Data Availability

All the data are available within the manuscript.
